# A New Normal: Integrating Lived Experience Into Scientific Data Syntheses

**DOI:** 10.3389/fpsyt.2021.763005

**Published:** 2021-10-29

**Authors:** Joanne R. Beames, Katarina Kikas, Maddison O'Gradey-Lee, Nyree Gale, Aliza Werner-Seidler, Katherine M. Boydell, Jennifer L. Hudson

**Affiliations:** Black Dog Institute, University of New South Wales, Sydney, NSW, Australia

**Keywords:** lived experience, integrative review, data syntheses, methodology, mental health, clinical psychology

## Introduction

Lived experience research in mental health incorporates the perspectives of people who live with mental health issues (1, 2). The integration of lived experience perspectives into the research process, from discovery to translation, has a long history, with seminal work in the mental health and clinical research fields emerging over four decades ago [e.g., (3–8)]. Recommendations and guidelines are now available that document how to include a breadth of perspectives and how to form advisory or participatory groups with appropriate representation of the target population (1, 9–11). There is no doubt that significant progress has been made to date to establish the value of lived experience research. One emerging area of integration is within traditional scientific data syntheses such as reviews and meta-analyses. This trend in methodology is moving slowly and has not yet taken hold in the status quo. In the current opinion piece, we identify this gap and build the case for appropriate integration of lived experience in future data syntheses. Such integration can support the identification and development of treatment approaches that align with the needs of those intended to use them.

## Rationale For Integration of Lived Experience

Systematic reviews and meta-analyses are considered the highest levels of scientific evidence. Researchers typically use their expertise to identify research questions and aims, review the literature according to set guidelines to ensure methodological rigor, and then make data-driven interpretations. The overall aim is to make conclusions about the state of evidence, identify practical implications, and offer future research directions. In the mental health and clinical field, recommendations may also be made about optimal approaches in clinical practice and service delivery (12, 13). These recommendations can be used to design new treatments or services, allocate funding, and influence policy decision making. Overall, research syntheses are routinely used for informing decision making about what should be done in the real world to help improve the lives of target groups in the community. Unfortunately, individuals with a lived experience of mental health are not typically involved in the underlying methodology. The implication is that decisions are being made without input from the individuals that have “insider information” and that stand to benefit (or be harmed) most.

It is estimated that 85% of funding for health research is “wasted” (14, 15). Research waste includes failure to publish research, unclear reporting of research, and poor study design and conduct (15, 16). Addressing research questions with limited relevance to clinicians, patients, and end-users is a key contributor to waste (16, 17). Incorporating lived experiences into mental health reviews and meta-analyses is therefore likely to improve the relevance and practical impact of the conclusions drawn. It is also well-aligned with integrated knowledge translation, which aims to meaningfully involve stakeholders from the outset of a research project with strong benefits in terms of research impact (18). Lived experience perspectives can be used to collaboratively identify gaps or problem areas, formulate research questions, interpret empirical findings, comment on empirical gaps, evaluate what implications mean for them and whether they are practically relevant, and help to form a judgment about whether future research on a topic is worthwhile (19). This integration could directly overcome limits of traditional approaches, which are often criticized for not representing the full scope of mental health and clinical research. Some scholars suggest that the hierarchy of evidence in evidence-based medicine privileges specific types of knowledge and devalues the lived experience voice (20).

There are many benefits to including lived experience perspectives in mental health research in general. These benefits include the production of higher-quality research, relevant outcomes with greater practical impact for the target population, increased likelihood that products or treatments will be accepted, increased trust in research and organizations, and increased empowerment and hope within those individuals who contribute (19, 21–24). Other benefits identified in the youth lived experience space include increased engagement, higher ethical standards, more insightful data analysis by translating meaning to adult researchers, and wider and more effective research dissemination and translation (10). Most evidence for the benefits of lived experience research is qualitative or based on informal retrospective accounts. However, the landscape is changing. More studies are quantitatively evaluating the outcomes and impact of lived experiences in health and social care research (25). For example, analysis of secondary data from the Mental Health Research Network (MHRN) portfolio database showed that studies with higher patient involvement were associated with achieving recruitment targets and with certain funding bodies [e.g., charity/non-for profits; (26)]. Outcomes and impact build the rationale for lived experience mental health research, and more work is needed to identify objective indicators for evaluation.

## Example of Integration

Systematic reviews and meta-analyses are not the only type of research synthesis methodology available. Integrative reviews are another alternative. These reviews allow for the combination of diverse methodologies (e.g., experimental and non-experimental research) to gather rich and nuanced information about a topic (27). An extension of this definition is to incorporate lived experience perspectives. Integration could occur along the whole review process, from problem identification, search strategy refinement, and interpretation of evidence. In addition to this, lived experience perspectives could be incorporated with the data synthesis results, allowing for contrast between findings, comment on gaps in empirical findings, and a judgment on value. This extension is not a new idea in and of itself, but it is not as widespread or given the same recognition as systematic approaches.

The Wellcome Trust is an influential global charitable foundation which has encouraged creative review methods to develop new insights into key problem areas in society. In 2020, the Trust established a worldwide effort to review the evidence on which aspects of interventions make a fundamental difference in preventing and treating youth anxiety and depression. Emphasis was placed on conducting integrative reviews that drew from broad data sources and placed lived experience perspectives at the center [e.g., (28, 29)]. Across the commissioned projects in 2020, research teams used unique applications of lived experience perspectives (30). This included involvement in making decisions about the project (e.g., research questions), defining the review process and reviewing the evidence, analysis and evidence synthesis, and reporting and dissemination. Incorporating lived experience perspectives at the outset of the integrative review process may facilitate more timely development of fit-for-purpose interventions.

## Significance of Integration

The integrative review methodology will challenge the status quo and change the key messages developed through research syntheses. For example, input from young people with lived experiences of mental health problems into research syntheses will lead to new areas of inquiry as well as different practical implications. Identifying and disseminating practical implications that are more relevant to the target audience, and align with their needs and preferences, is crucial for maximum impact at the individual and community level. It also has potential to lead to more effective knowledge translation strategies that can be applied in clinical practice and other community-facing services.

## Caveats of Integration

Lived experience research may lend itself to reviews in certain fields more than others. For example, mental health, clinical psychology, and clinical medicine are ideal candidates for application in the future. The aims of reviews and types of interpretations that ensue may also determine whether integration is appropriate. Recommendations that have direct relevance for individuals and groups in the community, for example those geared toward program development, service delivery, and clinical practice, may be more likely to align with the lived experience lens. The take home message is that systematic reviews should not necessarily replace integrative reviews, or vice versa, but there may be cases were one approach offers greater value-add.

With integrative methodology comes the issue of optimal balance between creativity and scientific rigor. The complexity inherent in combining diverse methodologies runs the risk of inaccuracy and bias. There is not yet a clear strategy to enhance and guide methods of analysis, synthesis, and conclusion drawing. A practical “how-to” guide would address this gap, outlining what can be done to take an inclusive approach that does not jeopardize quality, transparency, and replication of key research processes. Demonstrating how common barriers (e.g., time and resources) can be overcome to facilitate optimal engagement would be particularly worthwhile (21). Of relevance, the Australian National Health and Medical Research Council's quality improvement cycle for research describes how consumer participation can be integrated into all stages of research process (31, 32). These principles can be extended to apply to secondary data synthesis.

## Discussion

Lived experience research offers a wealth of rich information that can shape and enhance the quality and relevance of scientific reviews. Integrating these two seemingly separate research methodologies offers many benefits, not only for individuals with lived experience but also for the academic community. By ensuring that the research by scientists is relevant and endorsed by the people we aim to help, integration will increase the impact and translational capability of our findings. One example is the development and dissemination of psychological treatments that align with the needs and preferences of the target population. Creating a new normal by integrating lived experience into scientific data synthesis offers significant value and needs to be recognized as such to gain traction in the academic community. Best practice standards, organizational commitments, and resources are necessary to support this transition in the future.

## Author Contributions

JB conceptualized the project and wrote the manuscript. KK, MO'G-L, NG, AW-S, KB, and JH shaped the scope of the project. All authors have read, reviewed, refined, and approved the final manuscript.

## Funding

AW-S was supported by a NSW Health Fellowship. The funders had no role in the conceptualization of the perspective piece, writing the manuscript, or decision to decision to submit the paper for publication.

## Conflict of Interest

The authors declare that the research was conducted in the absence of any commercial or financial relationships that could be construed as a potential conflict of interest.

## Publisher's Note

All claims expressed in this article are solely those of the authors and do not necessarily represent those of their affiliated organizations, or those of the publisher, the editors and the reviewers. Any product that may be evaluated in this article, or claim that may be made by its manufacturer, is not guaranteed or endorsed by the publisher.
